# Beyond Gray Matter: White Matter Functional Network Signatures of Major Depressive Disorder Track Symptom Severity

**DOI:** 10.21203/rs.3.rs-11097214/v1

**Published:** 2026-10-14

**Authors:** Vince Calhoun, Malvika Sridhar, Bradley Baker

## Abstract

Resting-state neuroimaging studies in major depressive disorder (MDD) have consistently identified abnormalities across large-scale gray matter (GM) networks, including the default mode, salience, and sensorimotor systems. Although prior work has primarily focused on GM, evidence suggests that white matter (WM) hemodynamic signals are meaningful and reliable, supporting an active role for WM in brain function. A key gap remains in understanding WM functional network connectivity (FNC) and spectral characteristics in MDD. We used resting-state fMRI scans from the MDD-DIRECT phase II study, comprising 1,944 participants across 13 sites, to examine WM FNC and spectral characteristics in individuals with MDD and healthy controls using a spatially constrained independent component analysis (ICA) framework. This hybrid framework leverages ICA’s ability to identify spatially robust, functionally meaningful brain networks guided by replicable spatial priors to examine WM (1) FNC differences and (2) spectral power alterations between MDD and healthy participants. MDD was characterized by reduced long-range integrative connectivity alongside increased localized and limbic–sensorimotor coupling within WM networks, highlighting a systems-level imbalance in neural communication that may underlie core cognitive, affective, and somatic symptoms of depression. We also observed reduced low-frequency spectral power within frontal and subcortical WM systems involved in cognitive control, emotional regulation, and decision-making. Notably, both functional and spectral alterations were associated with clinical measures of depression and anxiety, with opposing associations observed for frontal connectivity patterns. Studying WM function alongside GM may provide a more comprehensive understanding of functional pathology in depression.

### Background

Major depressive disorder (MDD) is a debilitating neuropsychiatric disorder that is increasingly associated with large-scale brain network dysfunction. The resting-state functional magnetic resonance imaging (rsfMRI) literature has consistently identified abnormalities in gray matter networks, including the default mode, salience, and sensorimotor networks [1,2]. These findings have provided critical insights into neural mechanisms underlying depressive symptomatology and pharmacological effects and have informed the development of invasive and noninvasive brain stimulation strategies that target gray matter nodes within distributed brain networks [3,4]. Approximately 40% of the brain is made up of gray matter. The gray matter houses neuronal cell bodies (somas), dendrites, and axon terminals, playing a role in information processing, interpretation, and communication between regions [5]. While investigations of gray matter function have been crucial for our understanding of MDD, expanding to other brain tissues may reveal complementary aspects of network dysfunction.

Comprising nearly 60% of the human brain, the white matter offers a promising avenue for advancing a comprehensive understanding of MDD. The white matter consists of long, myelinated axons with lipoprotein-rich myelin sheaths that play a major role in long-distance signal transmission by increasing conduction velocity and enhancing fidelity of electrical signals [5,6]. White matter also exhibits a relatively higher glia-to-neuronal cell ratio, with a particular enrichment of oligodendrocytes [7]. Glial cells are the most abundant cell type in the central nervous system, performing a range of protective and supportive functions, including myelin production, indicating ongoing metabolic and structural maintenance. Interestingly, deeper white matter tissue also contains lesser-known neuronal cell bodies called interstitial neurons [8]. Overall, this supports the view that white matter is metabolically active and contributes dynamically to neural communication [9].

Much of the existing literature on white matter networks has focused on structural connectivity derived from diffusion magnetic resonance imaging (dMRI) tractography, which characterizes the structural integrity and maps white matter fiber pathways [10]. These approaches have been instrumental in identifying microstructural abnormalities in MDD and other psychiatric disorders [11,12]. Meta-analyses of dMRI studies have consistently identified decreased fractional anisotropy within cortical and subcortical regions of white matter fascicles connecting the prefrontal cortex [13,14]. However, structural connectivity alone does not capture the functional dynamics occurring within white matter pathways, suggesting that current analytical approaches may not fully capture its contributions to functional signals and network dynamics [15,16].

Emerging evidence indicates that blood-oxygenation-level-dependent (BOLD) signal fluctuations in the white matter contain meaningful and reliable functional information despite an overall weaker signal [9,17,16]. Functional imaging studies have even detected neuroplasticity within white matter tracts [18,19]. In a small-sample study using intracranial electrophysiology, white matter functional connectivity was correlated with electrophysiological activity across multiple frequency bands, suggesting that, like gray matter, white matter functional connectivity reflects the synchronization of underlying neural activity [20]. In rsfMRI, a high correlation between two white matter regions would indicate that their low-frequency BOLD fluctuations covary over time. Because white matter predominantly contains axons, this can be interpreted as reflecting synchronized axonal activity or metabolic demand along two communication pathways [21]. Recent work has shown that white matter exhibits organized functional networks at rest, with measurable static and dynamic connectivity that parallels the organization of gray matter networks [22,23]. These white matter networks appear to be robust and reproducible, often exhibiting greater temporal variability than gray matter networks, which suggests an active role in coordinating large-scale brain communication [16]. Notably, alterations in white matter functional organization have been shown to distinguish individuals with neurological and psychiatric disorders from healthy controls, underscoring their clinical relevance [24,25].

The spectral properties of white matter time courses also remain understudied, particularly in psychiatric illness. Spectral analysis provides complementary information by characterizing the frequency-specific composition of BOLD fluctuations, offering insight into whether disease-related alterations reflect changes in slow, persistent, faster, or variable oscillations [26]. Given evidence that white matter functional networks are highly dynamic and frequency-sensitive [27], examining spectral power alongside static functional connectivity may reveal abnormalities that are not captured by correlation-based measures alone.

Although research examining white matter function is limited, some functional imaging studies have found alterations in white matter functional connectivity and spectral properties. A study of adolescents with MDD identified reduced low-frequency power and altered local synchrony in the white matter, with abnormalities in callosal and projection fibers correlating with suicide risk [28]. Another study found disruptions in the functional coupling between gray and white matter networks, particularly within perceptual–motor and default mode systems, consistent with large-scale network decoupling in depression [29]. Randomized topology was observed in white matter connectomes, with clinical associations to symptom severity, enabling diagnostic classification [30].

In this study, we explore white matter functional network connectivity (FNC) and spectral differences between individuals with major depressive disorder and healthy controls using the REST-meta-MDD DIRECT Consortium dataset [31]. We utilize a spatially constrained independent component analysis (ICA) framework [32], leveraging its ability to identify spatially robust, functionally meaningful brain networks in a hybrid framework guided by replicable spatial priors [33], to support the exploration of 1) white matter FNC differences and 2) spectral power alterations within brain networks between MDD and healthy participants. We explore clinical associations of our neuroimaging findings with symptom severity of depression and anxiety as measured by the Hamilton Depression and Anxiety Rating Scales. Our goal is to leverage a combination of functional connectivity and spectral properties within white matter to capture complementary information regarding the organization and physiological functioning of white matter tracts in MDD that is not accessible through diffusion-based measures alone.

## Methods

### Dataset

We used resting-state fMRI scans from the MDD-DIRECT phase II study [31,34,35], a large-scale study that provided data access in accordance with the MDD-DIRECT phase II data-sharing plan. A total of 1,660 MDD patients and 1,341 healthy controls (age- and sex-matched) were scanned across 23 East Asian cohorts. The present study used data from a subset of 1,944 participants (1113 MDD patients and 831 healthy controls) from 13 sites with available sex, age, and site information, who were scanned on 3T MRI systems using 240 echo-planar imaging volumes (repetition time (TR) = 2000 ms; flip angle (FA) = 90°). MDD participants were diagnosed in accordance with the Diagnostic and Statistical Manual of Mental Disorders, 4th edition (DSM-IV). Informed consent was obtained from all participants, and the study protocols were approved by the relevant institutional review boards. Detailed site information is provided in Supplementary Table 1, and participant demographics are provided in Supplementary Table 2.

## Preprocessing

All scans from the MDD-DIRECT study were initially preprocessed using the Data Processing and Analysis for Brain Imaging on Surface (DPABISurf) software [36]. This included reference BOLD volume generation and skull-stripping with fMRIPrep [37], anatomical coregistration in Free Surfer [38], slice-timing correction with AFNI [39], head-motion parameter estimation performed using FSL [40], and spatial normalization to the Montreal Neurological Institute (MNI) 152 template. Additionally, spatial smoothing was performed using a 6-mm full-width, half-maximal Gaussian kernel on SPM12 [41]. Full pipeline details can be found in Chen et al. (2025) [31].

Our study employed spatially constrained ICA (sc-ICA) from the GIFT toolbox (https://trendscenter.org/software/gift/) [42] and focused on white matter functional connectivity analyses on the MDD Direct dataset. Our hybrid NeuroMark pipeline combines sc-ICA with replicable spatial priors derived from large, independent datasets comprising more than 100,000 scans [43]. These priors include 97 white matter intrinsic connectivity networks (ICNs) from multiple spatial scales previously organized and described in Itkyal et al. (2025) [33]. The 97 ICNs span 8 domains and 13 subdomains and are shown in Fig. 1. They are freely available in the Neuromark_fMRI_2.2_modelorder-multi in GIFT (also available separately at https://trendscenter.org/data/). Thus, 97 white matter-based spatial ICNs and their associated time series were derived for each subject using sc-ICA with the multi-scale Neuromark 2.2 template [44]. An overview of the methods pipeline is shown in Fig. 2.

### Static Functional Network Connectivity

The 97 ICA time series were then postprocessed, filtered to a 0–0.15 Hz frequency range, and white matter static functional network connectivity (sFNC) was computed using the Fisher Z-scored Pearson correlation coefficient (PCC), resulting in a 97x97 correlation matrix of functional network connectivity over the duration of the entire scan.

### Spectra

The fast Fourier transform (FFT) in GIFT on MATLAB was used to compute the time-series spectra for each white matter functional network in all subjects. Sixty-five evenly spaced frequency bins were defined within 0–0.2539 Hz, and statistical comparisons of power within each bin for each component were made, covarying for age, sex, and site. Multiple comparisons correction was applied across all frequency bins and functional networks.

### Statistical Analyses

All statistical comparisons of network connectivity, spectra, and their clinical associations with the Hamilton Depression (HAM-D) and Anxiety Rating Scales (HAM-A) [45,46] were conducted using ordinary least squares regression between MDD and healthy participants, covarying for age, sex, and site. Multiple comparisons were corrected for using Benjamini-Hochberg false discovery rate (FDR), and results with FDR $p < 0.05$ were deemed significant.

### Results

## Static functional network connectivity

Our analyses of white matter sFNC from resting-state fMRI data revealed significant diagnostic differences between individuals with MDD and healthy controls across 824 intrinsic connectivity network (ICN) pairs after FDR correction (Fig. 3). These differences reflected both increased and decreased functional connectivity across distributed large-scale networks, including frontal, paralimbic, temporal, sensorimotor, and subcortical thalamic and hippocampal regions.

### Associations of neuroimaging findings with depression and anxiety severity

Altered white-matter functional network connectivity across sensorimotor, frontal, insular, temporoparietal, and posterior hippocampal networks was significantly associated with depression severity (Fig. 4a). Positive associations were observed between middle sensorimotor and insular or anterior/posterior sensorimotor connections, as well as within frontal networks. Negative associations were prominent between frontal and temporoparietal or extended thalamic connections. Altered white-matter functional connectivity across frontal, cerebellar, insular, temporoparietal, and paralimbic networks was significantly associated with anxiety severity (Fig. 4b). Positive associations were observed between frontal and cerebellar or posterior hippocampal connections, and occipitotemporal and sensorimotor connections, whereas negative associations were prominent within frontal networks, as well as between paralimbic and thalamic connections.

### Spectral Analysis

Relative to healthy controls, individuals with MDD showed both increases and decreases in white matter spectral power, with most effects reflecting reduced low-frequency power in many white matter networks, predominantly the frontal, basal ganglia, insular, and occipitotemporal networks (Fig. 5d & 5e).

## Associations of neuroimaging findings with depression and anxiety severity

Reduced low-frequency power within the 0.0117–0.0156 Hz band in IC29 and IC43, constituting the posterior thalamic radiation and anterior corona radiata of the frontal network, was associated with clinical measures of depression severity, while reduced power within the 0.0234–0.0313 Hz band in IC25, constituting the left posterior limb of the internal capsule of the basal ganglia network, was associated with anxiety severity as measured by the HAMD and HAMA, respectively (Fig. 6).

### Discussion

MDD was associated with widespread alterations in sFNC of white matter networks during rest. These neuroimaging findings were further linked to clinical severity in depression and anxiety as measured by the HAM-D and HAM-A symptom scales, indicating that the pathology of MDD symptoms involves disruptions in the coordination of large-scale communication pathways that extend beyond traditional gray matter regions. Specifically, our results showed that individuals with MDD exhibited a strong decrease in connectivity across higher-order associative and limbic systems, intriguingly a three-way pattern of frontal–posterior hippocampus, insular/temporoparietal–frontal, and posterior hippocampus–insular/temporoparietal connections. This might imply reduced integration among networks that have been previously shown responsible for cognitive control, emotional regulation, and memory [47–50]. Reduced differentiation in the functional organization of white matter connectome gradients across fronto-limbic and thalamocortical tracts has been seen in individuals with MDD [51]. Recent structural network connectivity analysis identified distinct frontal–temporal connectivity patterns in MDD that vary based on comorbid anxiety status[52].

Our results also showed increased white matter connectivity within and between middle sensorimotor networks as well as between paralimbic–thalamic–hippocampal (PTH) networks. The PTH networks show overlap with the canonical limbic and Papez circuits responsible for emotion processing and regulation [53,54]. Moreover, in individuals with MDD, we observed that increased middle sensorimotor and PTH connectivity was associated with greater depression severity. This supports previous findings in the literature that have identified altered structural organization in the sensorimotor superficial white-matter system [55] and increased functional connectivity in anatomically proximal gray-matter sensorimotor networks [56] in MDD. Abnormal structural integrity within the Papez circuit has been previously related to symptoms of both depression and anxiety in adults with anxious MDD [57]. This suggests that our findings of similar alterations linked with depression severity may be due to maladaptive coupling between sensorimotor and affect regulatory networks. Reduced connectivity between frontal–temporal, frontal–cerebellar, and frontal–extended thalamic connections was also associated with greater depression severity. These findings are broadly consistent with prior evidence demonstrating altered white matter functional signaling in major tracts, including callosal, projection, and limbic-related pathways in depression, which have been linked to symptom burden and suicide risk [28].

Notably, while MDD individuals showed an overall lower within-network connectivity in frontal white matter pathways compared with healthy controls, within the MDD group, increased within-frontal connectivity was associated with greater depression severity (Fig. 4a). Prior reports of increased prefrontal connectivity in MDD have been linked to depressive rumination and self-referential processing (Luo et al., 2025; Myers et al., 2025). Our results, however, suggest that increased within-frontal connectivity may reflect a normative or adaptive pattern, but this greater connectivity scales with symptom severity in individuals with depression, potentially reflecting compensatory or maladaptive hyperconnectivity.

Significant associations were also found with symptoms of anxiety. Increased connectivity between frontal–cerebellar, frontal–occipitotemporal, occipitotemporal–sensorimotor, occipitotemporal–insular, and posterior hippocampal (specifically corona radiata)–insular regions was associated with greater anxiety severity (Fig. 4b). Reduced connectivity within frontal networks and between PTH connections was associated with greater anxiety, consistent with prior evidence of disrupted prefrontal regulatory control and altered limbic–thalamic integration in anxiety-related pathology [60].

Interestingly, the relationships between connectivity and clinical measures suggest partially opposing patterns in white matter functional network connectivity associated with depressive versus anxious symptom severity (Fig. 4c), despite both falling within the internalizing spectrum [61]. While depressive symptoms were associated with reduced connectivity between frontal–cerebellar brainstem and frontal–occipitotemporal regions, anxious symptoms showed positive associations with connectivity across these networks. Similarly, depressive symptoms were positively associated with frontal within-network and paralimbic–thalamic hippocampal connectivity, while anxious symptoms showed negative associations with these connections. These connectivity patterns may reflect differences in arousal states as proposed in the Tripartite model of anxiety and depression, consisting of general distress, anxious hyperarousal, and anhedonic depression [62]. Stronger coupling across frontal, occipitotemporal, and cerebellar systems may support heightened vigilance and externally oriented processing that characterizes anxiety, while reduced coupling across these systems may reflect diminished engagement with external stimuli and lower behavioral activation commonly observed in depression. Together, these findings demonstrate that MDD is characterized by reduced long-range integrative connectivity and increases in localized or limbic–sensorimotor coupling within white matter functional networks, highlighting a systems-level imbalance in neural communication that may underlie core cognitive, affective, and somatic symptoms of depression (e.g., altered large-scale network integration in MDD [49,52,54,55]. Furthermore, the identification of white matter functional and spectral alterations associated with symptom severity may provide a potential framework for linking neuroimaging markers of brain function to clinical phenotypes, with future implications for biomarker development and precision psychiatry approaches to treatment.

The results of our spectral analyses showed that individuals with MDD primarily exhibited decreased power at low frequencies (< 0.04 Hz) in the frontal, basal ganglia, insular, and occipitotemporal white-matter networks. Studies of gray-matter networks have identified a similar decrease in the amplitude of low-frequency fluctuations (ALFF) in frontal, paralimbic, and temporal regions [63,64].

Importantly, reduced power in the ~ 0.01 Hz range in posterior thalamic radiations and anterior corona radiata (superior and anterior corona radiata, superior fronto-occipital fasciculus, anterior limb of internal capsule, external capsule, corpus callosum, and superior longitudinal fasciculus) of the frontal network was associated with clinical measures of depression severity (Fig. 6). Similarly, reduced power in the ~ 0.025 Hz range in the left posterior limb of the internal capsule (superior corona radiata, superior longitudinal fasciculus, and external capsule) of the basal ganglia network was associated with anxiety severity (Fig. 6). Interestingly, these regions align closely with a 2024 study that identified decreased ALFF in white matter regions, including the corpus callosum, left posterior limb of the internal capsule, and the posterior and superior corona radiata, in MDD, and found these regions to be negatively correlated with the suicide scale [28]. Taken together, reduced low-frequency activity in frontocortical and basal ganglia white matter pathways is associated with greater clinical severity.

Spectral findings indicate disruption of low-frequency dynamics within frontal white matter systems that support executive control and thalamocortical integration, as well as within basal ganglia circuits involved in goal-directed behavior and emotional regulation [65,66]. These patterns are consistent with dysfunction in core regulatory systems implicated in MDD [67,68]. Clinically, these results suggest the potential relevance of interventions targeting large-scale network dynamics, including pharmacological neuromodulation approaches, rather than localized mechanisms.

The current study is not without its limitations. First, limited access to detailed cognitive and behavioral measures constrained our ability to link alterations in connectivity to specific symptom domains or functional impairments. Second, the use of a binary MDD diagnosis precluded examination of depression subtypes and more fine-grained symptom dimensions, which may show distinct connectivity profiles. Third, the geographically restricted sample, while reducing within-study variability, may limit generalizability, as cultural and regional factors can influence symptom expression, reporting, and diagnostic practices. Finally, the unimodal imaging design limits the ability to directly relate white matter functional connectivity findings to complementary structural or electrophysiological measures.

Despite these limitations, this study has several strengths, including a large sample size, a data-driven ICA approach, a relatively underexplored investigation of white matter functional connectivity and spectral properties in depressive disorder, and the use of validated clinical measures to link neuroimaging findings to symptom severity. Our findings highlight the importance of studying white matter function alongside gray matter to better understand the clinical features of depression and anxiety. Future research should investigate specific symptom dimensions of MDD, such as anhedonia, insomnia, psychomotor retardation, and others, which may reveal unique white matter profiles that could aid biomarker development.

### Declarations

## Acknowledgment

This work was supported in part by the NIH R01EB006841, R01MH123610, and NSF 2316421. We gratefully acknowledge the MDD-DIRECT Phase II study investigators and participants for their contributions to the study and for making these data available for the present research[31,34,35].

## Author Information

### *Affiliation*

Center for Translational Research in Neuroimaging and Data Science (TReNDS), Georgia State University, Atlanta, GA

Malvika Sridhar, Bradley Baker, Vince D. Calhoun

### *Consortia*

**The DIRECT Consortium**

*Contributions*

M.S.: analysis; visualization; writing—original draft, literature review, and editing. B.B.: data preprocessing, writing—editing. V.D.C.: supervision; interpretation; writing—critique and editing.

### *Corresponding author*

Correspondence to Vince D. Calhoun: vcalhoun@gsu.edu

## Additional Information

### *Competing interests*

The authors declare no competing interests.

### *Ethics declarations*

This study was conducted in retrospect using data collected from human participants in compliance with all relevant ethical standards.

## Data availability

Data are available through the MDD-DIRECT Phase II data-sharing process, subject to its data-sharing plan and applicable access requirements.

## Figures

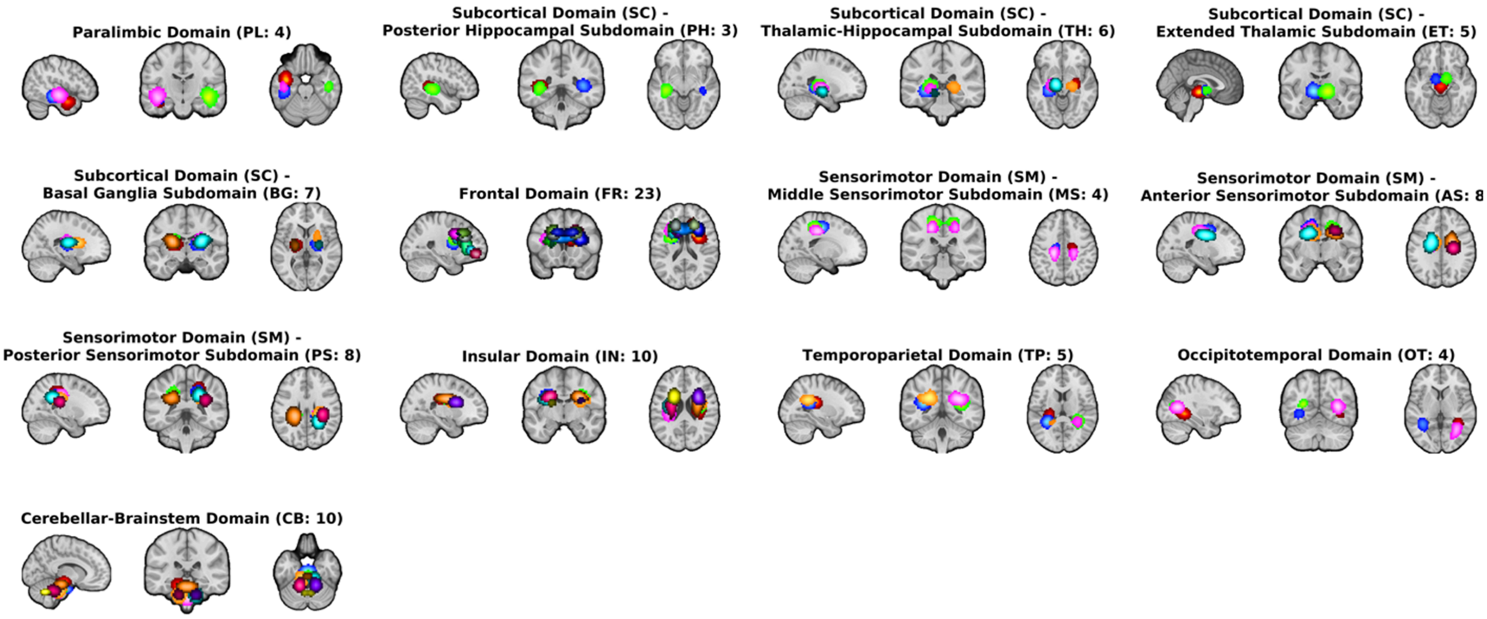

### Figure 1

**Overlaid spatial maps of the 97 intrinsic connectivity networks (ICNs) from the NeuroMark 2.2 multi-scale template are plotted above.** Image from Itkyal et al. (2025) [33], under a Creative Commons license CC BY-NC-ND 4.0 29. paralimbic (PL), subcortical-posterior hippocampal (SC-PH), subcortical-thalamic hippocampal (SC-TH), subcortical-extended thalamic (SC-ET), subcortical-basal ganglia (SC-BG), frontal (FR), sensorimotor-middle sensorimotor (SM-MS), sensorimotor-anterior sensorimotor (SM-AS), sensorimotor-posterior sensorimotor (SM-PS), insular (IN), temporoparietal (TP), occipitotemporal (OT), and cerebellar-brainstem (CB)

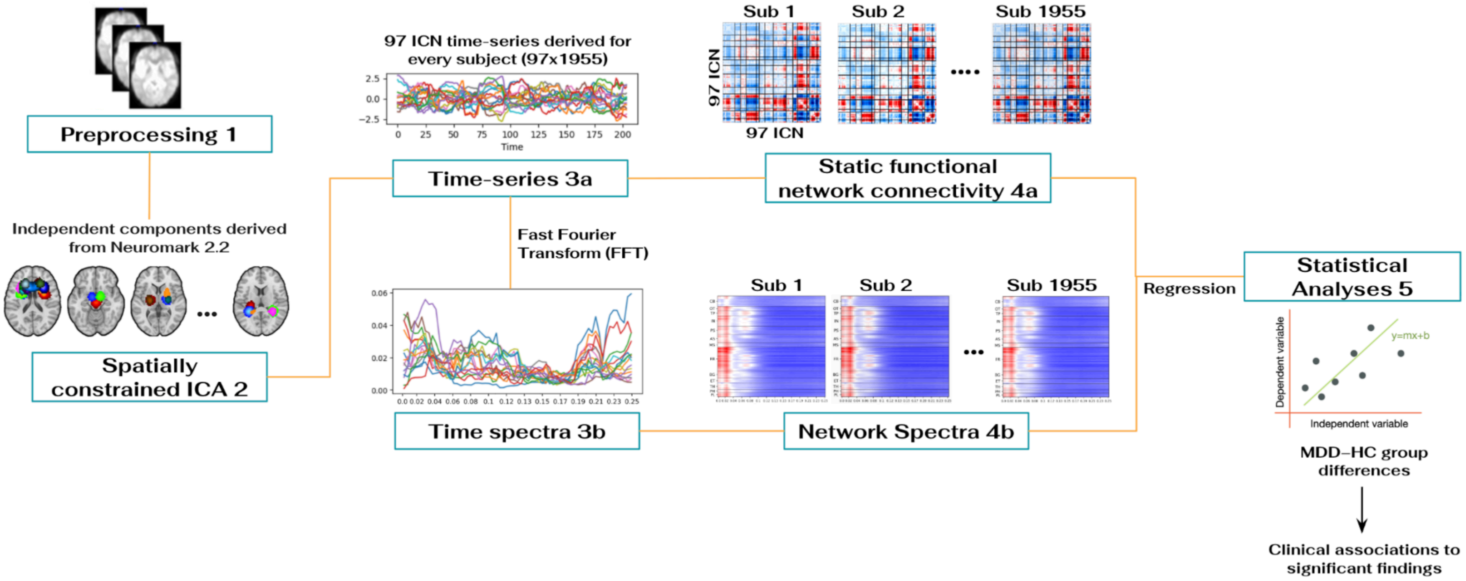

### Figure 2

### Methods Pipeline

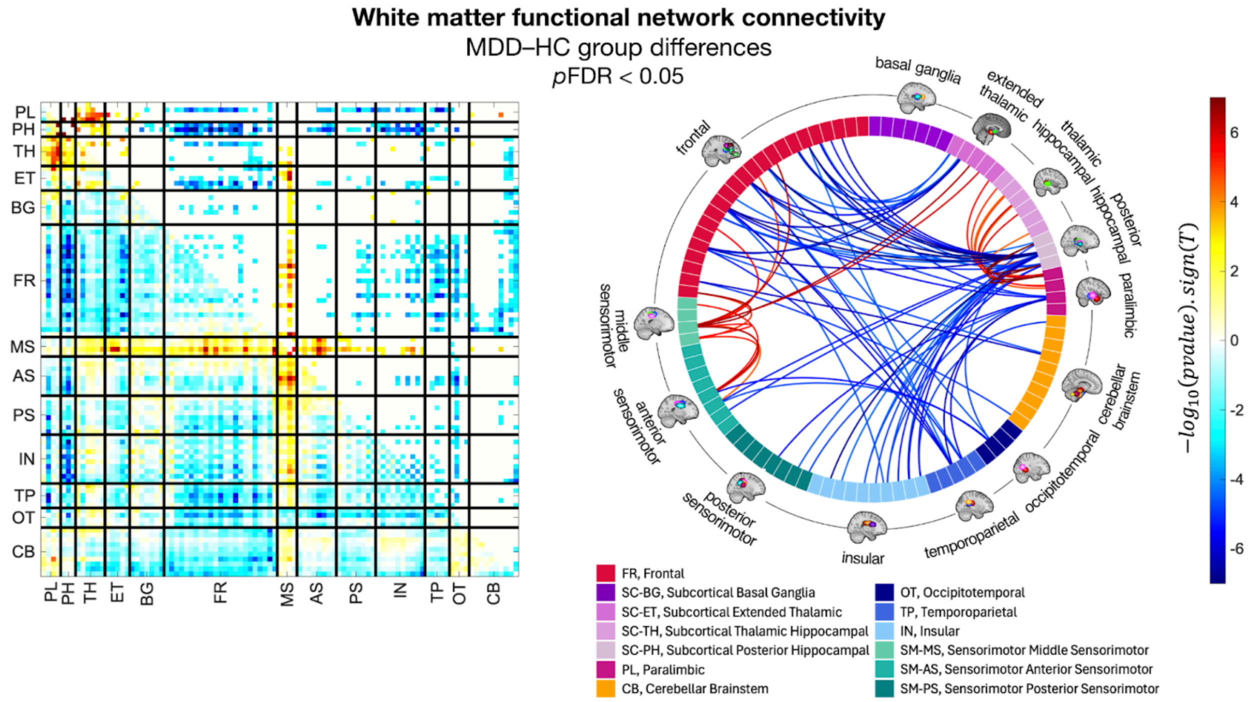

### Figure 3

(left) Lower triangle displays uncorrected differences (MDD−HC) in functional network connectivity for all ICN pairs, while the upper triangle displays 824 FDR-thresholded significant network pairs (pFDR <

0.05). (right) Circular connectome shows the top 10% (83) of significant pairs. In terms of connectivity, *red=MDD>HC, blue=MDD<HC. Abbreviations: FDR, false discovery rate; ICN, intrinsic connectivity network; MDD, Major Depressive Disorder*

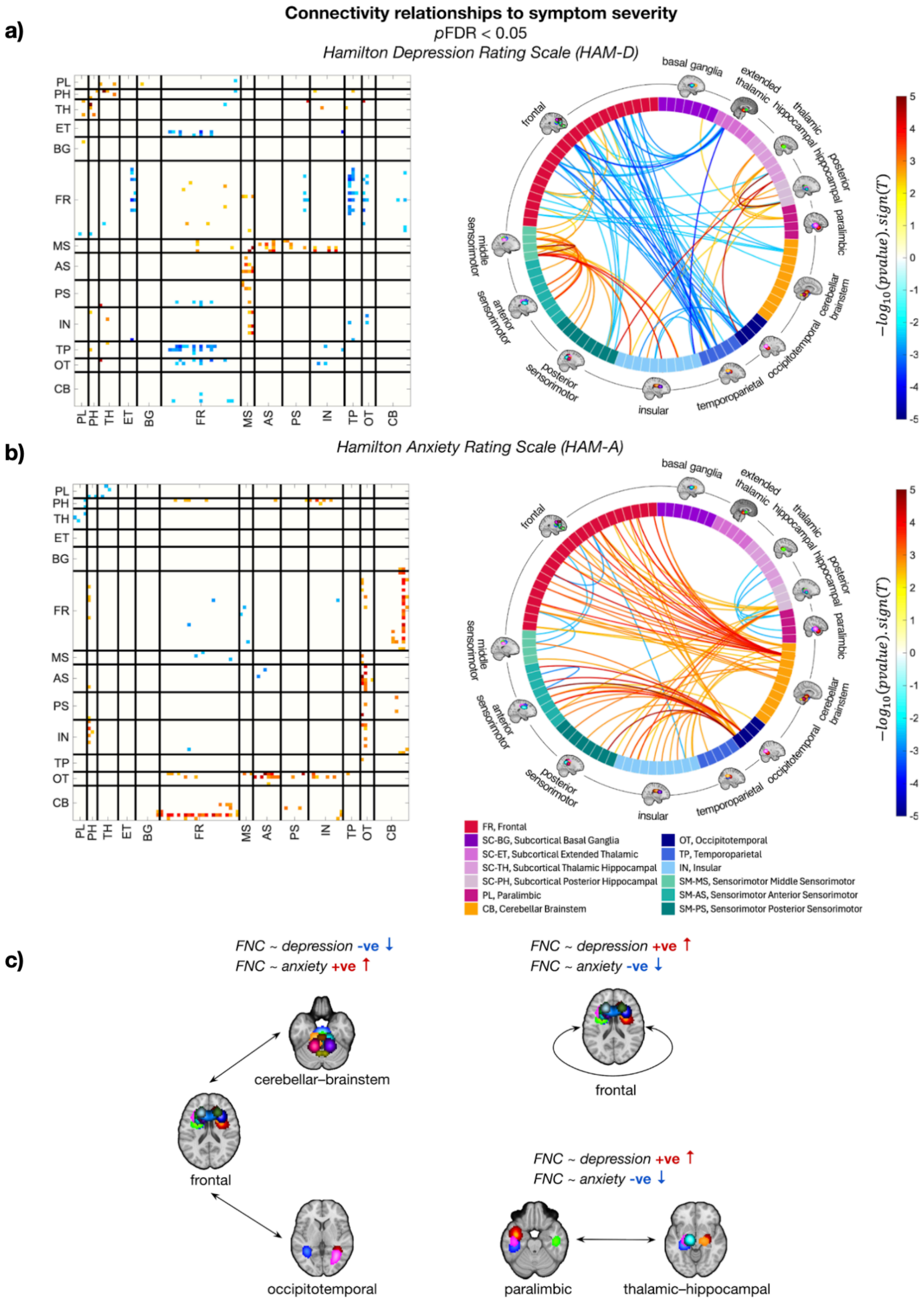

### Figure 4

**The matrices and connectomes show FDR-thresholded network pairs significantly associated (pFDR < 0.05) with diagnostic symptoms in the MDD group**: a) Depression Scale HAMD (92 ICN pairs; N=934), b) Anxiety Scale HAMA (79 ICN pairs; N=670). c) White matter functional network pairs that display opposing connectivity patterns between depression and anxiety severity: frontal–cerebellar brainstem and frontal–occipitotemporal (left), frontal–frontal (top right), and paralimbic–thalamic hippocampal (bottom right). *Abbreviations: FDR, false discovery rate; HAM-A, Hamilton Anxiety Rating Scale; HAM-D, Hamilton Depression Rating Scale; ICN, intrinsic connectivity network; MDD, Major Depressive Disorder*

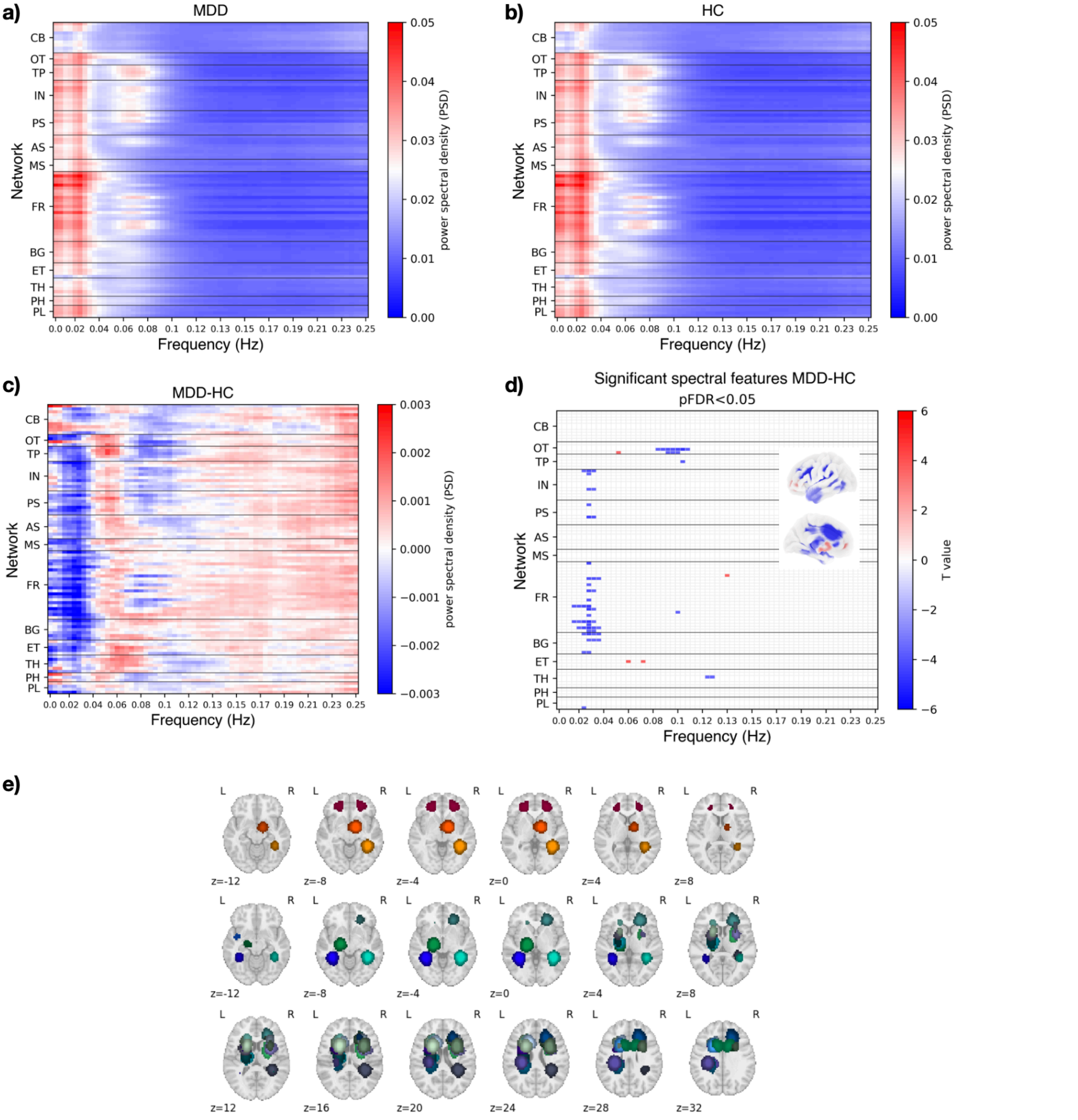

### Figure 5

a) Mean spectral power distribution across 97 ICNs in individuals with MDD. b) Mean spectral power distribution across 97 ICNs in healthy controls. c) Difference in mean spectral power between MDD participants and healthy controls. d) FDR-thresholded significant differences in network spectral power within specific frequency bands between MDD and healthy controls. e) Composite map of significant ICNs with spectral power differences in specific frequency bands, MDD>HC (top row), MDD<HC (bottom two rows). *Abbreviations: FDR, false discovery rate; ICNs, intrinsic connectivity networks; MDD, Major Depressive Disorder*

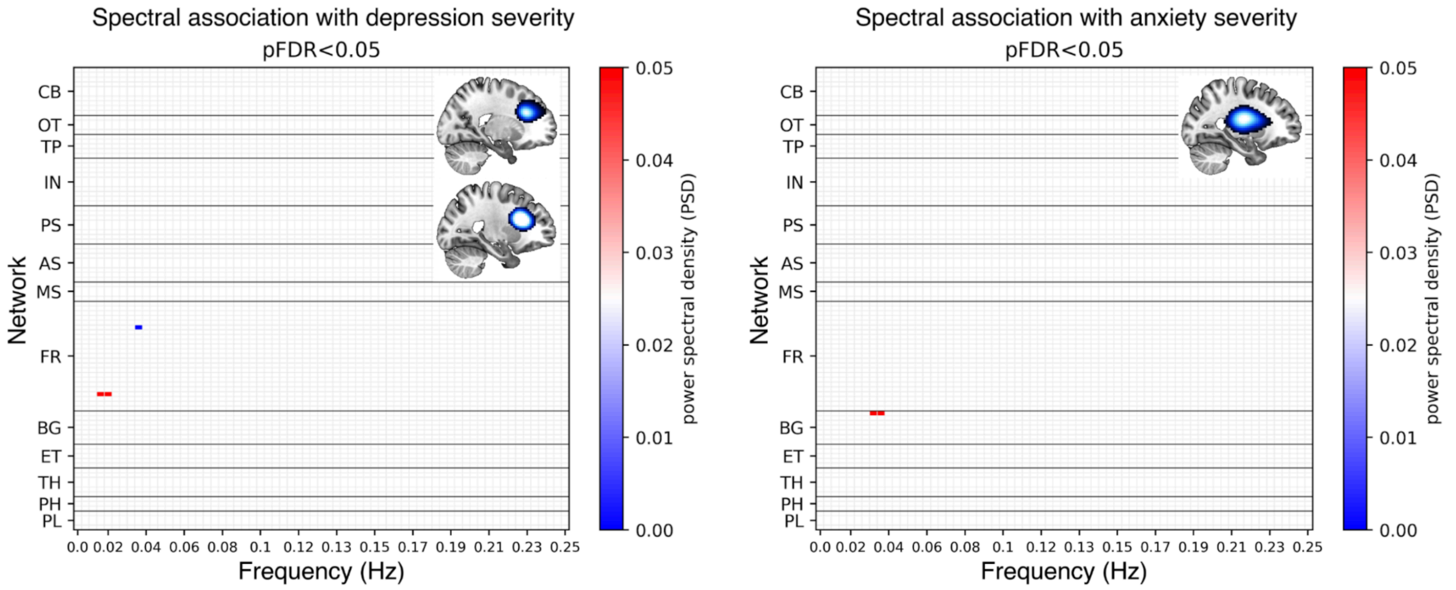

### Figure 6

**The matrices show FDR-thresholded ICN pairs that were significantly associated with diagnostic symptoms in the MDD group (pFDR < 0.05), depression scale HAMD (left), anxiety scale HAMA (right).** *Abbreviations: FDR, false discovery rate; HAM-A, Hamilton Anxiety Rating Scale; HAM-D, Hamilton Depression Rating Scale; ICN, intrinsic connectivity network; MDD, Major Depressive Disorder.*
